# Hirudin inhibits glioma growth through mTOR‐regulated autophagy

**DOI:** 10.1111/jcmm.17851

**Published:** 2023-08-04

**Authors:** Ying Ma, Senbin Wu, Fanyi Zhao, Huifeng Li, Qiaohong Li, Jingzhi Zhang, Hua Li, Zhongmin Yuan

**Affiliations:** ^1^ Department of Neurology Institute of Neuroscience, Key Laboratory of Neurogenetics and Channelopathies of Guangdong Province and the Ministry of Education of China, The Second Affiliated Hospital, Guangzhou Medical University Guangzhou China; ^2^ Guangdong Province Key Laboratory of Brain Function and Disease Guangzhou China; ^3^ Department of Traditional Chinese Medicine The Second Affiliated Hospital of Guangzhou Medical University Guangzhou China; ^4^ Laboratory animal center, The Second Affiliated Hospital Guangzhou Medical University Guangzhou China; ^5^ Guangdong‐Hong Kong‐Macao Greater Bay Area Center for Brain Science and Brain‐Inspired Intelligence Guangzhou China

**Keywords:** apoptosis, autophagy, glioma, Hirudin, mTOR

## Abstract

Glioma is the most common primary malignant brain tumour, and survival is poor. Hirudin has anticancer pharmacological effects through suppression of glioma cell progression, but the molecular target and mechanism are poorly understood. In this study, we observed that hirudin dose‐ and time‐dependently inhibited glioma invasion, migration and proliferation. Mechanistically, hirudin activated LC3‐II but not Caspase‐3 to induce the autophagic death of glioma cells by decreasing the phosphorylation of mTOR and its downstream substrates ULK1, P70S6K and 4EBP1. Furthermore, hirudin inhibited glioma growth and induced changes in autophagy in cell‐derived xenograft (CDX) nude mice, with a decrease in mTOR activity and activation of LC3‐II. Collectively, our results highlight a new anticancer mechanism of hirudin in which hirudin‐induced inhibition of glioma progression through autophagy activation is likely achieved by inhibition of the mTOR signalling pathway, thus providing a molecular basis for hirudin as a potential and effective clinical drug for glioma therapy.

## 1 INTRODUCTION

Glioma is the most common and malignant primary tumour among central nervous system cancers and has a very poor prognosis in patients. Despite advances in surgery, radiotherapy, chemotherapy, targeted therapy and stem cell therapy, the treatment outcomes in glioma patients remain very poor, with an average survival time of approximately 14–17 months, not to mention the patients' pain and heavy economic burden.^1^, ^2^, ^3^ Therefore, new treatments with greater therapeutic efficacy and fewer side effects are urgently needed as adjuvant therapies for glioma.

Hirudin, a direct thrombin inhibitor, is a polypeptide isolated from the salivary gland of the medicinal leech Hirudo medicinalis and is composed of 64–66 amino acids with a molecular weight of approximately 7000 Da; however, with the development of bioengineering technology, the synthetic recombinant hirudin (rH), lacking tyrosine residues sulfated at site 63, possesses similar pharmacological activity to natural hirudin, but with a lower thrombin inhibition constant, which is why we chose natural hirudin and the chemical structure of natural hirudin is shown in Figure S1 ^4^. Originally recorded in Shennong's Classic of Material Medical, leeches have the efficacy of breaking blood, expelling stasis and freeing collateral vessels in traditional Chinese medicine (TCM).^4^ Currently, hirudin has been used widely in the clinic as an anti‐coagulation and anti‐thrombus drug by potently affecting the steps of the intrinsic coagulation caused by thrombin.^5^ In addition, it was recently reported that hirudin also exhibits excellent anticancer activities in the treatment of various tumours, including human glioma,^6^ non‐small cell lung cancer^7^ and bladder cancer.^8^ Most interestingly, there is a strong positive correlation between thrombin and malignant glioma,^9^, ^10^, ^11^ and the inhibition of thrombin by hirudin greatly suppresses the growth of glioma cells in vitro^6^. While hirudin appears to be a promising strategy for inhibiting the progression of human glioma, the mechanism and application remain unclear.

Recently, exploring the function of autophagy in malignant progression and response to treatments has been an interesting focus in many types of cancer. Autophagy, as a mechanism for maintaining cellular homeostasis, is an intracellular lysosomal degradation process^12^ and is upregulated when stimulated by extracellular or intracellular stress to cause irreversible damage, which leads to apoptosis or necrosis.^13^ Large numbers of studies have demonstrated that there is a negative correlation between autophagy activation and glioblastoma progression,^14^, ^15^, ^16^ and the induction of lethal autophagy in tumour cells has been implicated in the anticancer effects of a variety of drugs. In addition, temozolomide, as a primary clinical chemotherapeutic drug for human glioma, has also been proved to play a critical role in anti‐glioblastoma therapies via autophagy induction.^17^, ^18^ Consequently, autophagy is taken as a potential therapeutic target into consideration for both glioma prevention and therapy, whereas its role and mechanisms require further clarification. Recently, a number of studies have shown that downregulation of the pro‐survival mTOR pathway can prevent autophagosome fusion to lysosomes at later time points to induce the autophagic death of glioblastoma cells.^19^, ^20^, ^21^ In addition, transcriptome‐based network analysis and follow‐up experimental research have shown that the expression of autophagy‐related pathway proteins, such as mTOR, was significantly reduced by hirudin treatment in rats with unilateral ureteral obstruction.^22^ However, whether the inhibitory effect of hirudin on human glioma involves autophagy has not been demonstrated.

In this study, we investigated the hirudin‐induced autophagy effect in antiglioma progression and the underlying mechanism. Using three kinds of glioma cell lines, we observed that hirudin induces autophagy activation by upregulating the expression of LC3‐II in glioma cells. Further study showed that hirudin caused a decrease in the phosphorylation of mTOR and its downstream factors ULK1, P70S6K and 4EBP1 and preliminarily showed an inhibitory effect of hirudin on glioma via cell‐derived xenografts (CDXs) in nude mice in vivo. Our findings provide a new molecular theoretical basis for hirudin to become a potential therapeutic drug for glioma.

## 2 MATERIALS AND METHODS

### 2.1 Antibodies and reagents

The following antibodies were purchased from Cell Signalling Technology: Caspase‐3 (9662), cleaved‐Caspase3 (9661), p‐ULK1 (14202), ULK1 (8054), p‐P70S6K (9234), P70S6K (2708), p‐4EBP1 (2855). LC3 (14600‐1‐AP), P62/SQSTM1 (18420‐1‐AP), p‐mTOR (67778‐1‐Ig), mTOR (66888‐1‐Ig), 4EBP1 (60246‐1‐Ig), GAPDH (60004‐1‐Ig) were purchased from Proteintech. Secondary antibodies used include Goat Anti‐Mouse IgG (Jackson 115–035‐146), Goat Anti‐Rabbit IgG (H + L) (Jackson 111–035‐045). Hirudin were purchased from Guangxi Keyken Technology Group Co., Ltd. (Guangxi, China).

### 2.2 Cell culture and treatment

Human glioma cell lines LN229, U251, U87 MG were purchased from the Chinese Academy of Sciences Cell Bank (Shanghai, China) and National Infrastructure of Cell Line Resource, cultured as described previously.^23^ Cells were cultured in whole media containing DMEM supplemented with 10% fetal bovine serum (Hyclone) and 1% penicillin–streptomycin (10,000 U/mL, Invitrogen, USA) in 5% CO2 in a humidified incubator at 37°C. All cell lines were regularly checked for mycoplasma contamination.

### 2.3 Cell viability and proliferation assay (CCK‐8 assay)

To detect cell viability, cells were pre‐seeded into 96‐well plates at 4 × 10^4^ per well in 150 μL media. After the cells were treated with hirudin at different concentrations or times, 10 μL of CCK‐8 reagent (Beyotime Biotechnology, C0039) was added to each well and incubated for 0.5 h to 1 h at 37°C. Absorbance was measured at 450 nm using a light absorption microplate reader (Molecular Devices, SpectraMax 340PC384). Cell numbers were calculated based on a standard curve derived from serial cell dilution.

### 2.4 Wound healing assay

LN229, U251 and U87 MG cells were cultured in 6‐well plates. Scratch wounds were generated with a new 20 μL pipette tip when cells grew to approximately 90% confluence. The cells were then washed with PBS twice and incubated in low‐serum medium, while the treatment group was treated with 6 U/mL hirudin prepared with low‐serum medium, for 24 h. Pictures were taken under a microscope. ImageJ software was used to quantify the gap distance. The relative wound healing area was calculated according to the formula by referring to the literature^24^:

Relative wound healing area = (A_0_ ‐ A_24_) / (B_0_‐B_ 24 _).

A: Hirudin treatment, A_0,_ A_24_ is the scratch area at 0 h, 24 h, respectively.

B: Hirudin‐free treatment, B_0,_ B_24_ is the scratch area at 0h, 24h, respectively.

The values shown are the means of three wells from three independent experiments.

### 2.5 Cell migration and invasion assay (Transwell assay)

In the cell migration and invasion test, LN229, U251 and U87 MG cells were placed in 24‐well transwell chambers (8 μm pore size, Corning). The cell invasion assays were performed using transwell chambers that were precoated with Matrigel diluted with serum‐free DMEM, while the cell migration assay was performed without Matrigel. The cells in the treatment group were pretreated with 6 U/mL hirudin for 4 h, and then the cells in each group were inoculated with approximately 1 × 10^5^ cells in 200 μL medium containing 1% fetal bovine serum in the upper chamber. The lower chamber was filled with 600 μL medium containing 20% fetal bovine serum. They were then incubated at 37°C with 5% CO_2_ for 16 h, fixed in 4% paraformaldehyde for 15 min, and then stained in 0.05% crystal violet prepared with methanol diluted with PBS for 30 min. The cells were gently wiped from the upper part of the filter using a cotton swab, the filter was allowed to dry naturally and the cells from the lower part of the filter were checked and counted under a microscope.

### 2.6 Western blotting analysis

Cell lysates were prepared and Western blotting was performed as described previously.^3^, ^23^ Briefly, whole cell lysates were separated by SDS‐polyacrylamide gel electrophoresis (SDS–PAGE) and transferred to a polyvinylidene fluoride membrane (Millipore Corporation). All membranes were probed with primary antibodies at 4°C overnight, followed by incubation with the secondary antibod. The primary antibodies used are listed in the Antibodies and Reagents as before. ECL Reagent was used to develop the intensity of the immunoblotting band.

### 2.7 Immunohistochemistry (IHC)

The tumour tissues were embedded in paraffin, sliced into 5 mm sections, and incubated with primary antibody LC3 (1:400; #14600‐1‐AP, Proteintech) at 4°C overnight, rinsed with PBS and incubated with horseradish peroxidase‐linked goat anti‐rabbit secondary antibody. DAPI (1:10,000) was added to stain the nuclei, 5 min at RT. The imaged under a light microscope (Olympus) and the staining intensity and the expression levels were evaluated according to the German immunohistochemical score.^3^, ^25^

### 2.8 Tumour xenografts and evaluation of the antitumor effect in vivo

All experimental protocols for tumour xenografts were approved by the Animal Care and Use Committee of Guangzhou Medical University (A2021‐009, A2022‐053). Five‐week‐old male BALB/c nude mice (Laboratory Animal Center of Southern Medical University) were divided into three groups: the vehicle, medium‐dose hirudin and high‐dose hirudin groups. Each group of seven mice was bred in the Laboratory Animal Center under specific pathogen‐free conditions. Subcutaneous glioma tissue was constructed in CDX nude mice as previously describe^23^. The equivalent drug dose conversion ratio between animals and humans (9.1: 1) was calculated by referring to the literature^26^ and the method showing in the website (https://www.docin.com/p‐246618839.html). In brief, the daily intake of hirudin in adults (500 U/60 kg means 1250 mg Hirudin per 60 kg body weight) was converted into the middle‐dose (On average, about 2 U/mL per nude mice) of animal treatment, and the double dose was the high‐dose (4 U/mL per nude mice) of treatment. CDX nude mice in the treatment group were intraperitoneally injected with 2 U/mL or 4 U/mL hirudin as described previously,^27^ and those in the vehicle group were injected with the same dose of physiological saline solution. The treatments were administered every day for 21 days. Each group was observed daily and all tumours retrieved from CDX nude mice were measured and recorded. Relative tumour volume (RTV) was calculated according to the formula:

V = (length width width)/2

where length represents the longest diameter and width represents the shortest diameter.

### 2.9 Statistical analysis

The data were expressed as mean ± standard deviation (SD) and represent the results of at least three independent experiments. Statistical significance was determined using the unpaired *t*‐test for two‐group experiments. Comparisons were analysed with one‐way analysis of variance (anova) or two‐way anova with selected pairs analysis. P value lower than 0.05 were considered statistically significant and were indicated as follows: *p < 0.05; ** p < 0.01; *** p < 0.001.

## 3 RESULTS

### 3.1 Hirudin inhibits glioma cell viability in a concentration‐ and time‐dependent manner

**FIGURE 1:**
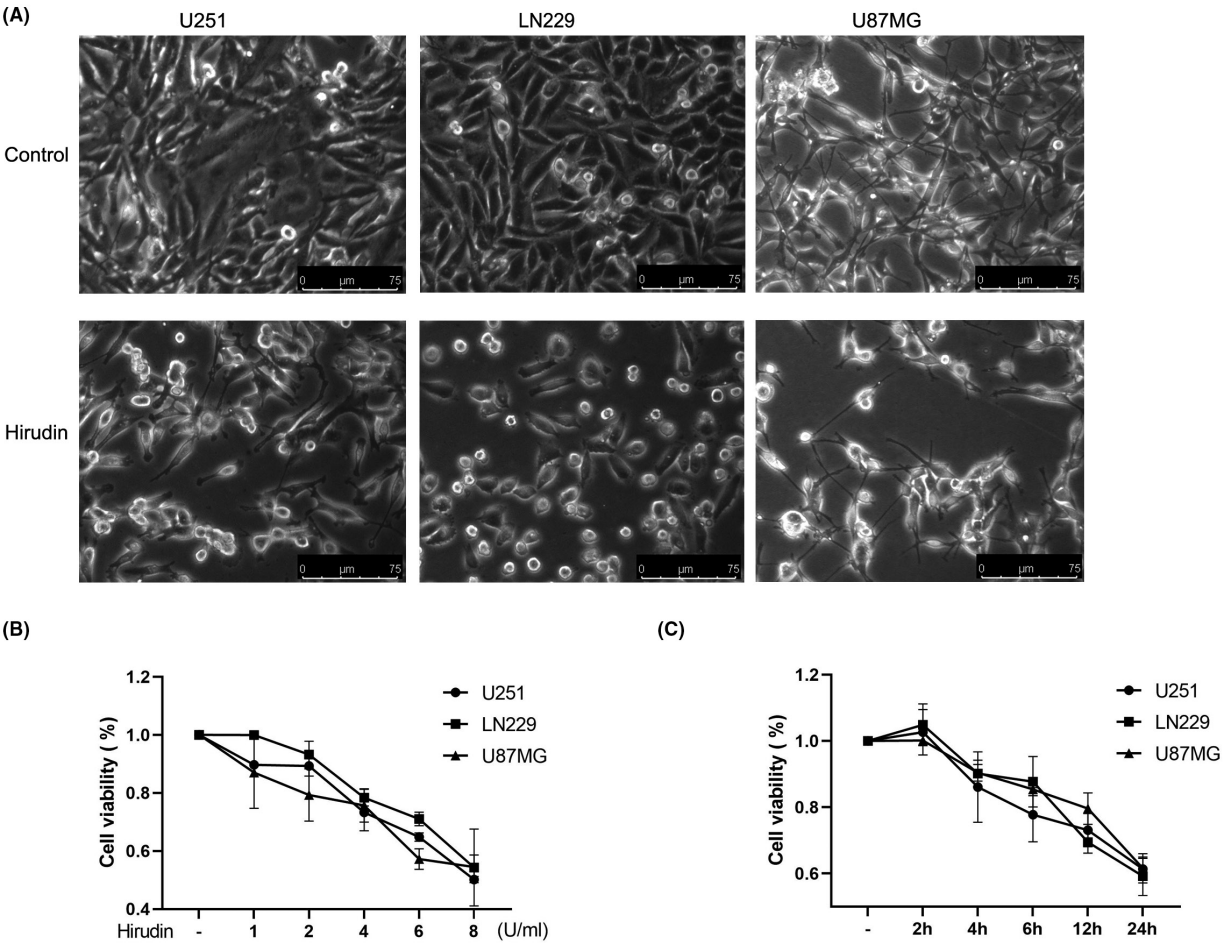
Hirudin inhibits glioma cell viability in a concentration‐ and time‐dependent manner. (A) Three glioma cell lines U251, LN229 and U87MG were treated with Hirudin, and their morphology was observed. (Scale bar =75 μm). (B) U251, LN229 and U87MG cells were treated with hirudin at concentrations of 1 U/mL, 2 U/mL, 4 U/mL, 6 U/mL, and 8 U/mL, and CCK8 assays were performed to determine the proliferation rates at each concentration. The data are presented as the mean ± SD, n = 3. (C) U251, LN229 and U87MG cells were treated with 6 U/mL concentrations of hirudin for 2, 4, 6, 12 and 24 h, and CCK8 assays were performed to determine the proliferation rates at each time point. The data are presented as the mean ± SD, *n* = 3.

We treated three glioma cell lines, U251, LN229 and U87MG, with hirudin and observed the cell morphology and the dynamic correlation between cell viability and the concentration and time course of hirudin treatment. Treating cells with hirudin for 12 h induced a robust inhibition of U251, LN229 and U87MG cells, with the cells becoming bright and round, decreasing in number and shortening the length of the pseudopodia as observed from cell morphology (Figure 1A), suggesting that glioma was efficiently suppressed by hirudin, which is consistent with previous reports^6^. CCK8 assays demonstrated that compared with cells without any treatment, hirudin treatment significantly inhibited the proliferation of U251 and LN229 cell lines from the treatment concentration of 1 U/mL, whereas the treatment concentration of 2 U/mL began to inhibition of the proliferation of U87MG cells, and the treatment concentrations of 6 U/mL and 8 U/mL showed a stronger inhibitory effect. Calculating the data on the Image J software, the half maximal inhibitory concentration (IC_50_) of hirudin on U251, LN229 and U87MG cell lines were 7.23 U/mL, 7.64 U/mL and 5.68 U/mL respectively (Figure 1B). In addition, based on the half maximal inhibitory concentration (IC_50_) data of three cell lines, we treated them with hirudin at a concentration of 6 U/mL, which remarkably inhibited the proliferation of two cell lines, LN229 and U87MG, starting at 4 h posttreatment, while inhibition of U251 started at 6 h posttreatment, and all induced greater suppression lasting up to 12 and 24 h in all three cell lines (Figure 1C). Taken together, these results indicate that hirudin substantially reduces cellular viability and proliferation in glioma cells, which is positively correlated with the time and concentration of treatment.

### 3.2 Hirudin inhibits the proliferation, migration and invasion of glioma cells

**FIGURE 2:**
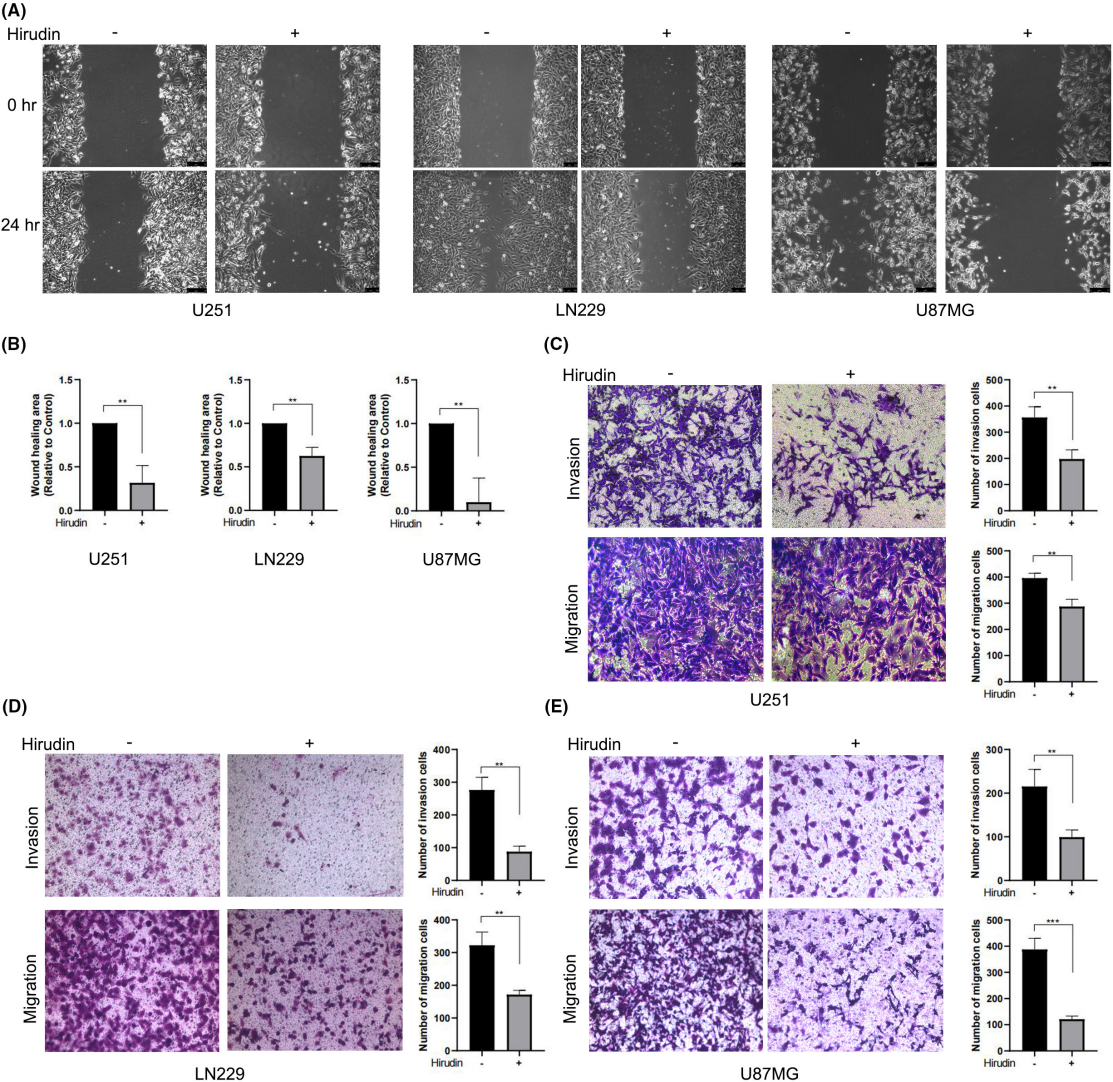
Hirudin inhibits the proliferation, migration and invasion of glioma cells. (A) A scratch‐wound assay was used in U251, LN229 and U87 MG cells, with or without hirudin treatment, and then their proliferation, migration and wound healing were observed at 0 and 24 h. (B) The wound healing areas were analysed using ImageJ 1.8.0, and compare wound healing areas at 0 and 24 h post‐scratch to perform equal conversion using hirudin‐free treatment results as a reference to calculate wound healing percentage. The data are presented as the mean ± SD, *n* = 3, t‐test analysis, **p < 0.01. (C–E) Transwell assays were used in three glioma cell lines that were pretreated with or without hirudin, and their migration and invasion were then observed. Photoshop CC2020 to count the number of cells and GraphPad Prism 8 was used for mapping and statistical analysis. The data are presented as the mean ± SD, *n* = 3, t‐test analysis, **p < 0.01, ***p < 0.001.

To further clarify the effect of hirudin on the proliferation, migration and invasion of glioma, we performed scratch‐wound assays and transwell assays using three glioma cell lines, U251, LN229 and U87MG. Hirudin has been shown to inhibit the proliferation, migration and invasion of glioma cells. Scratch‐wound assays demonstrated that U251, LN229 and U87MG cells at the wound edge polarized and migrated into the wound space within 24 h; however, hirudin inhibited this migration (Figure 2A) and the wound healing area treated with hirudin was significantly better than that without hirudin (Figure 2B), which suggests that the proliferation, migration and wound healing of glioma cells were efficiently suppressed by hirudin. Furthermore, the transwell assay also demonstrated that the cell migration and invasion functions of the three glioma cell lines were significantly inhibited after pretreatment with 6 U/mL hirudin compared with the cell lines without hirudin pretreatment (Figure 2C–E). Taken together, these results indicate that hurudin treatment causes an inhibition of proliferation, migration and invasion in glioma cells.

### 3.3 Hirudin induces autophagy‐dependent growth arrest but not apoptosis in glioma cells

**FIGURE 3:**
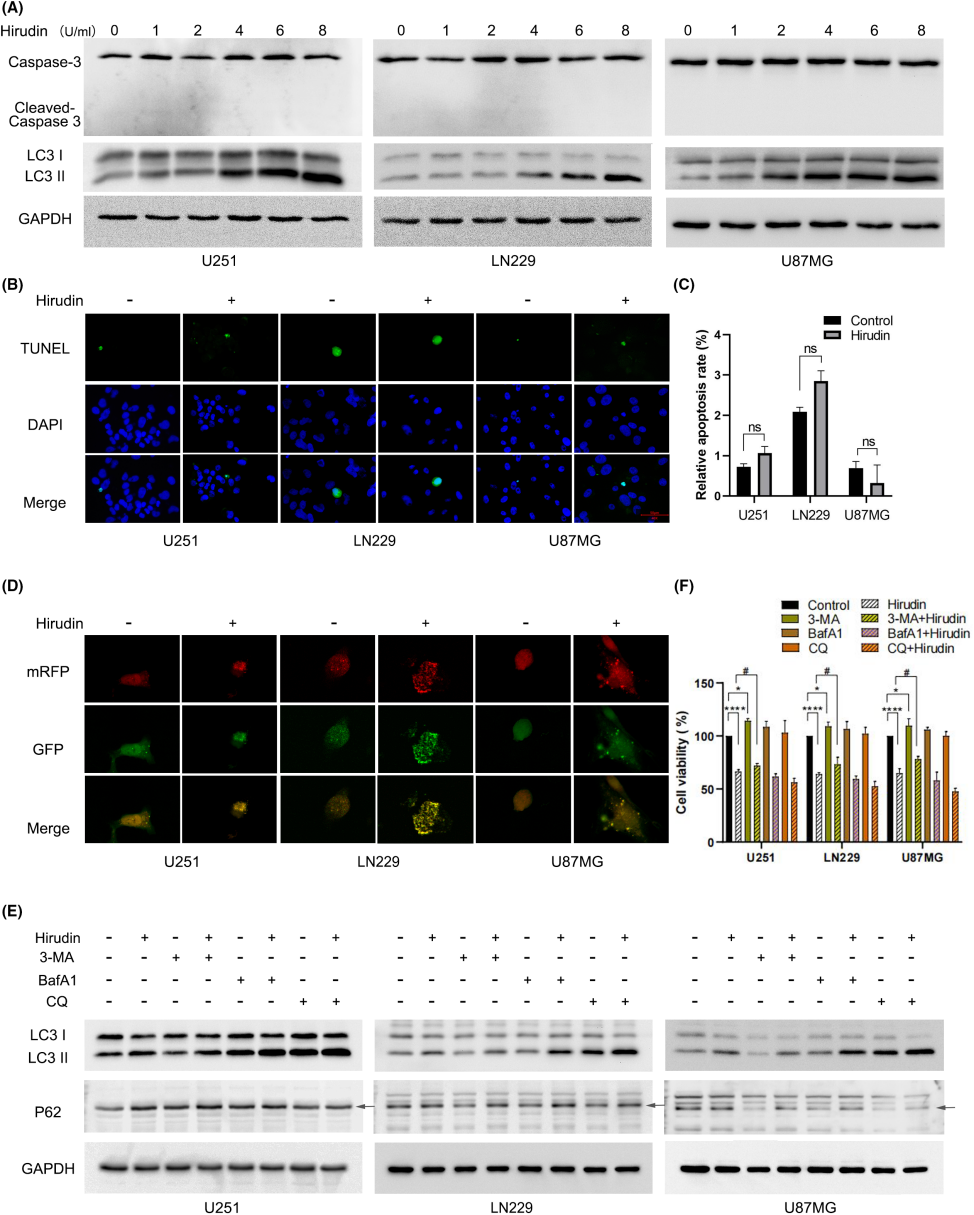
Hirudin induces autophagy‐dependent growth arrest but not apoptosis in glioma cells. (A) U251, LN229 and U87MG cells were treated with hirudin at concentrations of 0 U/mL, 1 U/mL, 2 U/mL, 4 U/mL, 6 U/mL, and 8 U/mL for 12 h, and WB was performed to detect the expression of Caspase‐3, Cleaved‐Caspase 3 and LC3I/II at each concentration. GAPDH was probed to verify equal loading. (B, C) TUNEL assay was used to detect the apoptosis rate of three glioma cell lines treated with or without hirudin. The data are presented as the mean ± SD, n = 3, t‐test analysis, ns, non‐significant. (Scale bar =50 μm). (D) mRFP‐GFP‐LC3 plasmid transfected U251, LN229 and U87MG cells for 48 h, followed by hirudin (6 U/mL) treatment for 12 h, photographed by confocal laser microscopy. The green puncta represented autophagosomes, and the red puncta represented autolysosomes. (E) U251, LN229 and U87MG cells were treated with autophagy inhibitor and/or hirudin for 12 h, western blot analysis was performed to detect the expression of the LC3I/II and P62/SQSTM1. GAPDH was probed to verify equal loading. (F) The autophagy inhibitor 3‐MA, BafA1, and CQ were used in the CCK8 assays to elucidate the exact role of hirudin in the autophagic activity of glioma cells. The data are presented as the mean ± SD, *n* = 3, one‐way anova, *, compared to control group, *p < 0.05, ****p < 0.0001; ^#^, compared to hirudin group, ^#^ *p* < 0.05.

Cell death includes apoptosis, autophagy, and necroptosis and other forms. To further explore the pathway by which hirudin inhibits the proliferation, invasion and migration of glioma cells, we detected whether the autophagy core protein LC3 and apoptosis executor protein Caspase‐3 were altered in three glioma cell lines following hirudin treatment. As shown in Figure 3A, hirudin treatment at different concentrations of 1 U/mL, 2 U/mL, 4 U/mL, 6 U/mL and 8 U/mL for 12 h in U251, LN229 or U87MG cells did not cause the expression of Cleaved‐Caspase3, but accompanied by the continuous increase of LC3‐II expression, a marker of autophagy initiation. The terminal deoxynucleotidyl transferase‐mediated dUTP nick end labeling (TUNEL) assay also showed that there was no significant difference in measured apoptotic cell numbers between hirudin treatment and control (Figure 3B,C).

To monitor the autophagy procession posttreatment of hirudin, we transfected three glioma cell lines with mRFP‐GFP‐LC3 plasmid, an artificial indicator widely used to assess the autophagy flux. The results showed that hirudin treatment could increase the numbers of phagosome‐ or autophagosome‐like puncta compared with control in the three cell lines (Figure 3D). However, we noted that hirudin‐caused puncta are yellow, resulted from the red‐green overlap, but not red with GFP quenching, suggesting hirudin probably cause a functional obstacle of the late‐stage autophagy, besides initiating autophagy. Similar phenomenon was observed in the previous report in which DMAMCL induced an autophagy‐dependent inhibition of glioma cell proliferation.^28^

To further get the evidences that hirudin regulates autophagy procession, the three glioma cell lines were treated with hirudin or in combination with the inhibitors for early‐ or late‐stage autophagy, including 3‐methyladenine (3‐MA), Bafilomycin A1 (BafA1) or chloroquine (CQ) and following western blot analysis was performed to examine the changes of LC3‐II and p62/SQSTM1, an indicator of the late stage autophagic flux which is normally degraded by the lysosomal proteases through the interaction with LC3‐II in the late stage of autophagy. As shown in Figure 3E, hirudin‐induced LC3‐II expression could be significantly rescued by 3‐MA due to the inhibition of the early‐stage autophagy, while BAFA1 or CQ greatly augmented LC3‐II levels via blocking the function of the later‐stage autophagy and hirudin plus BAFA1 or CQ had additive effects. Furthermore, compared with the vehicle control, hirudin treatment caused a robust increase of p62/SQSTM1 in the three cell lines, and 3‐MA obviously rescued the effects. Although BAFA1 or CQ treatment affected p62/SQSTM1 expression in a cellular context, but hirudin in combination with each inhibitor consistently caused an accumulation of p62/SQSTM1. The results suggested that hirudin induces an autophagy disorder not only through promoting autophagy initiation but obstructing the late‐stage autophagy flux.

To further observe if hirudin‐induced cell growth arrest is through autophagy, the three glioma cell lines were treated with hirudin alone or in combination with the used autophagy inhibitors and following cellular viability was assessed. As shown in Figure 3F, 3‐MA treatment alone promoted the viability of the glioma cells and hirudin‐mediated cell growth inhibition was significantly rescued by 3‐MA, but BAFA1 and CQ had no such effects.

Taken together, the results indicated that hirudin induces autophagy‐dependent growth arrest in glioma cells, but not apoptosis.

### 3.4 Hirudin suppresses the activity of mTOR signalling in glioma cells

**FIGURE 4:**
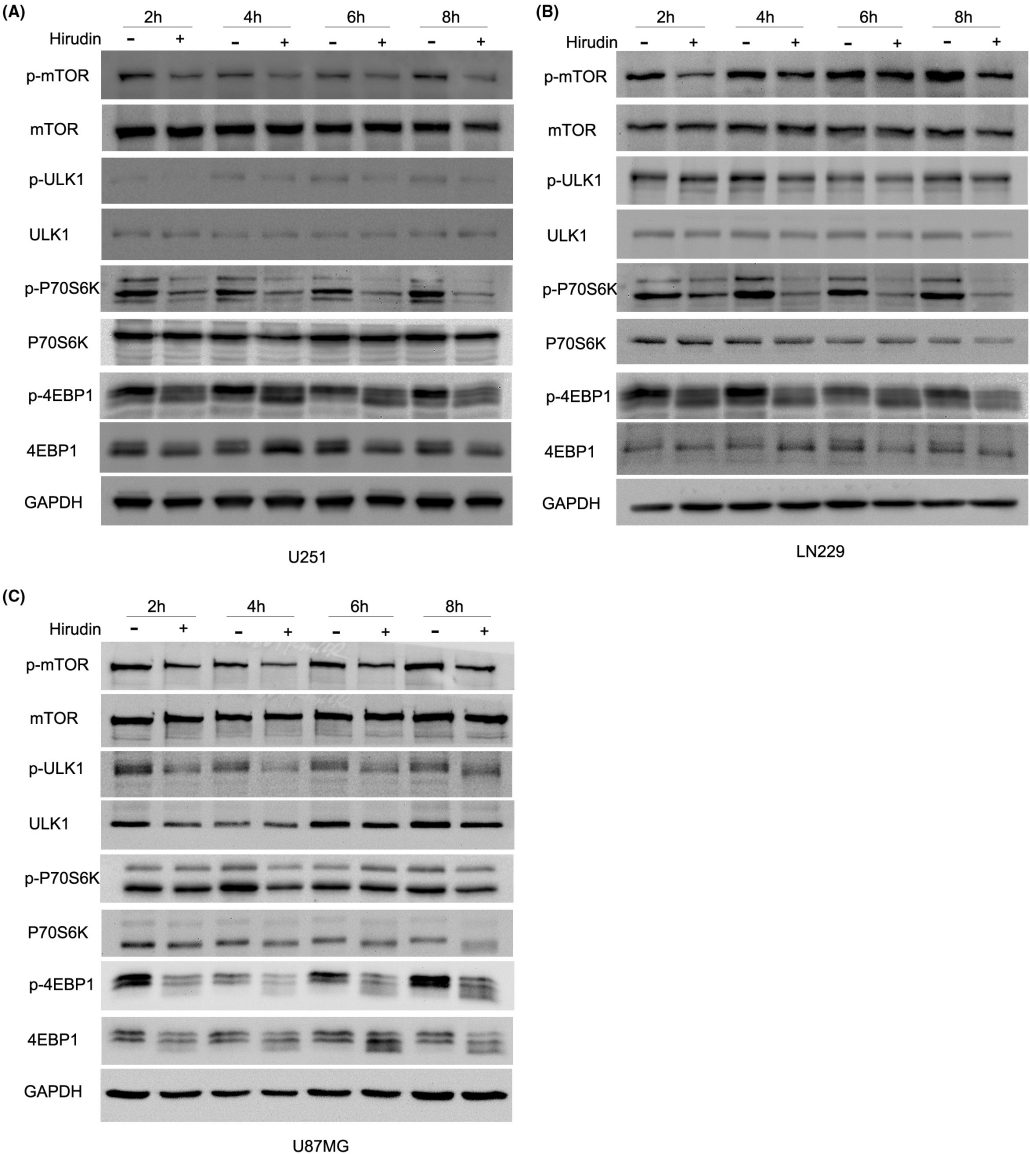
Hirudin suppresses the activity of mTOR signalling in glioma cells. (A–C) U251, LN229 and U87MG cells were treated with hirudin for 2 h, 4 h, 6 h and 8 h, and western blot analysis was performed to analyse the protein and phosphorylation levels of mTOR, ULK1, P70S6K, and 4EBP1. GAPDH was probed to verify equal loading.

A low level of basal autophagy is likewise crucial to sustain the cellular homeostasis, where it is well established that activated mTORC1 inhibits autophagy induction by direct phosphorylation of ULK1 at several serine residues. mTORC1 inactivation by nutrient deprivation or other stresses, in turn leads to immediate de‐repression of ULK kinase activity and thereby activates the transition of LC3‐I to LC3‐II to promote autophagy induction and maturation.^29^ We then explore whether hirudin‐induced autophagy in glioma cells is associated with the activity of the mTOR signalling pathway by detecting the phosphorylation levels of ULK1, P70S6K, and 4EBP1, which are the core proteins of the mTOR signalling pathway. Western blot analysis showed that compared with the control, mTOR phosphorylation was sharply inhibited by the application of hirudin to U251, LN229 and U87MG cells, starting at 2 h posttreatment and lasting to 8 h. As a consequence, hirudin treatment resulted in a remarkable decrease of phosphorylated ULK1, P70S6K, and 4EBP1 (Figure 4A–C). The results suggest hirudin induces an autophagy in glioma cells through mTOR inactivation.

### 3.5 Hirudin inhibits the tumour growth via inducing autophagy in U87MG‐derived xenograft mice

**FIGURE 5:**
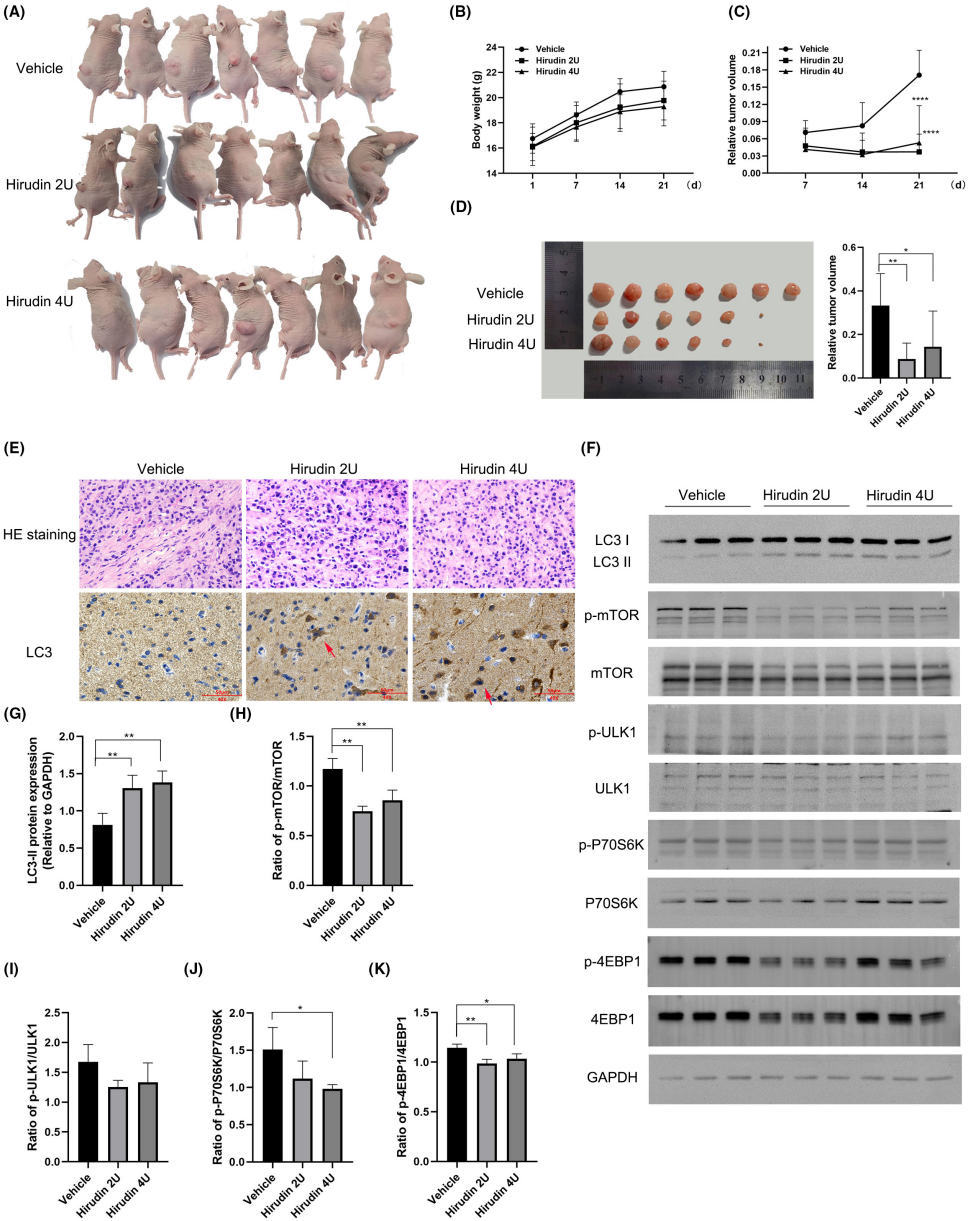
Hirudin inhibits the tumour growth via inducing autophagy in U87MG‐derived xenograft mice. (A) Images of tumours of posttransplantation CDX animal models treated by intraperitoneal injection with or without hirudin for 21 days. (B) The weight of posttreatment CDX animal models with or without hirudin. The data are presented as the mean ± SD, *n* = 7, two‐way anova, there is non‐significant. (C) Subcutaneous glioma volume of posttreatment CDX animal models with or without hirudin. The data are presented as the mean ± SD, *n* = 7, two‐way anova, ****p < 0.0001 (hirudin 2 U or hirudin 4 U vs. vehicle). (D) Glioma tissue volume images of CDX animal models after treatment. The data are presented as the mean ± SD, *n* = 7, one‐way anova, *p < 0.05, **p < 0.01 (vehicle vs. hirudin 2 U vs. hirudin 4 U). (E) Haematoxylin–eosin staining revealed the necrotic region in the subcutaneous glioma tissue of CDX nude mice in each group and conventional‐immunohistochemistry analysis of LC3 expression was performed in all groups. (Scale bar =50 μm). (F) CDX nude mice were treated with hirudin, and then western blot analysis of LC3I/II expression was performed. Similarly, proteins of mTOR signalling pathway were also analysed. Subsequent statistical analysis of LC3‐II levels (G) and autophagy‐related pathways proteins phosphorylation levels (H–K) compared with the vehicle group were also performed. The data are presented as the mean ± SD, *n* = 3, one‐way anova, *p < 0.05, **p < 0.01.

CDX models were established in nude mice by subcutaneously implanting U87MG cells. According to the conversion results of human and animal dosages, medium‐dose hirudin (2 U per CDX mice) or high‐dose hirudin (4 U per CDX mice) was injected intraperitoneally to further examine the inhibitory effect on glioma growth in vivo. It was worth noting that neither obvious subcutaneous bleeding, urinary and faecal bleeding nor internal bleeding in brain, liver, intestine or other parts was found during the whole experiment process (Figure S2). By monitoring the body weight and glioma growth of CDX nude mice daily, we observed that compared with the vehicle group, the subcutaneous glioma body in the CDX nude mice grew slowly following treatment with two doses of hirudin (Figure 5A). Hirudin did not cause significant changes in the body weight of mice compared with the vehicle (Figure 5B), but can greatly reduce the volume of subcutaneous glioma (Figure 5C).

On the 21st day of the experiment, the subcutaneous glioma tissue of CDX nude mice was removed, and it was observed that the glioma volume treated with medium‐dose or high‐dose hirudin were far smaller than that of the vehicle group (Figure 5D). Subsequently, HE staining indicated that the necrotic areas were significantly reduced in CDX mice after hirudin treatments compared to the vehicle group. In addition, the immunohistochemistry (IHC) results showed that LC3‐positive IHC staining in the glioma tissue of CDX nude mice treated with hirudin was stronger than that in the vehicle group (Figure 5E). Similarly, we reconfirmed that the level of LC3‐II expression was greatly increased following hirudin treatments while suppressing the expression of p‐mTOR, p‐ULK1, p‐P70S6K and p‐4EBP1 (Figure 5F–K). Overall, these results indicate that hirudin treatment significantly inhibit the growth of subcutaneous glioma in CDX nude mice by inducing autophagy.

## 4 DISCUSSION

**FIGURE 6:**
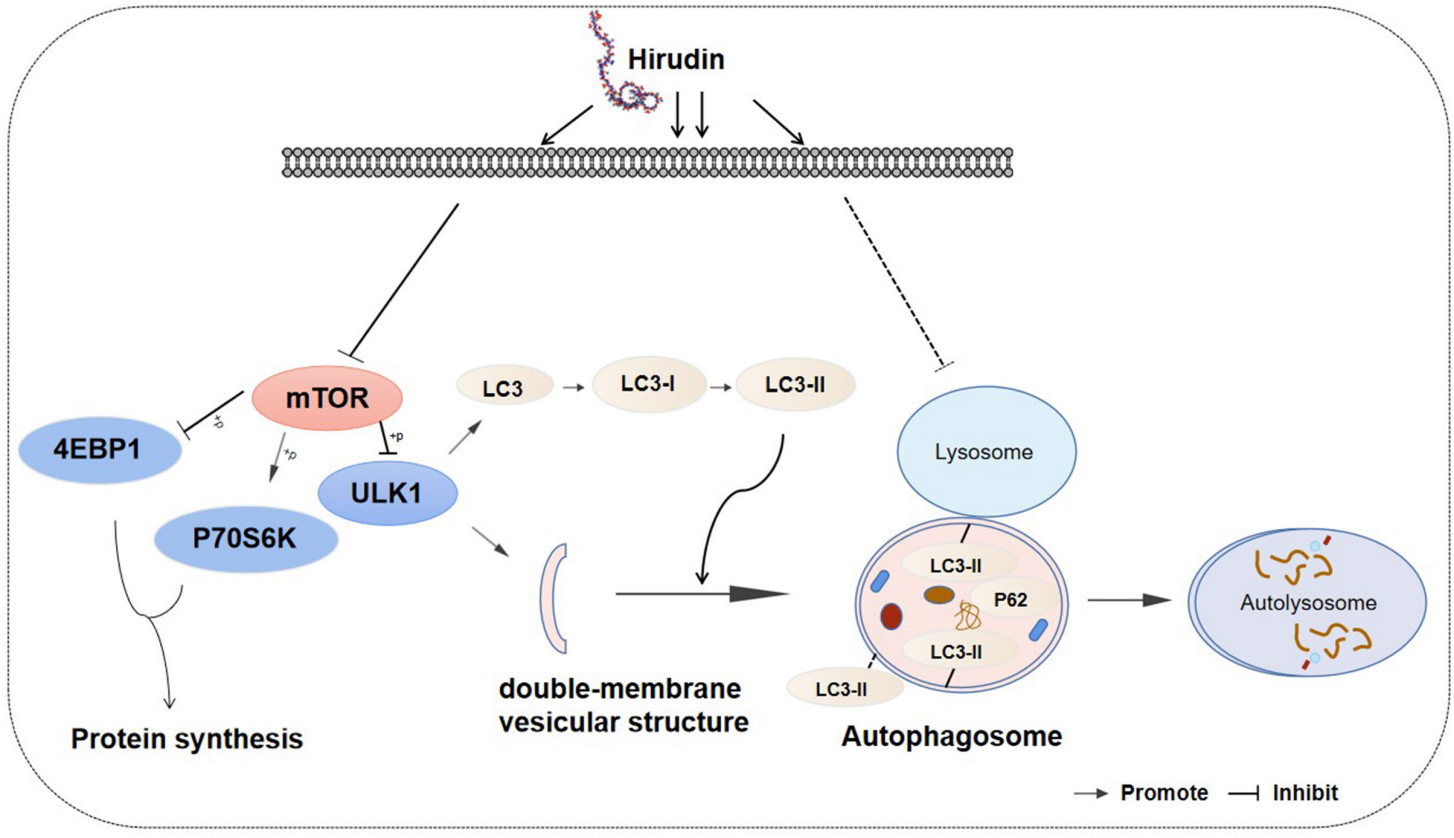
The schematic diagram of hirudin‐mediated autophagy disorder. For one thing, hirudin treatment significantly decreases the activity of mTOR signalling pathway, which results in an initiation of autophagy progression at early stage by increasing LC3‐II expression. For another, hirudin treatment affects the function of the late stage of autophagy flux with an obstacle in LC3‐II transition and p62/SQSTM1 degradation.

In this study, we demonstrate that hirudin suppresses glioma cell growth without through a typical apoptosis induction, as no significant increases in caspase 3 activation and TUNEL staining‐positive numbers at posttreatment. Instead, hirudin treatment significantly decreases the activity of mTOR signalling pathway, which results in an initiation of autophagy progression at early stage by increasing LC3‐II expression and the puncta‐like phagosome and autophagosome numbers. Further, hirudin treatment also affects the function of the late stage of autophagy flux with an obstacle in GFP quenching and p62/SQSTM1 degradation. Our in vitro and in vivo results highlight the potential of hirudin as a novel therapeutic drug against glioma by triggering autophagic cell death modes (Figure 6).

Different tumour therapy may induce cancer cell death via diverse mechanisms and the ultimate goal is to kill cancer cells. Conventional cancer therapy with therapeutic drugs usually evokes cell death by inducing apoptosis. However, many types of cancer including glioblastoma commonly has a highly deregulated genome thus with highly frequent deletion of tumour suppressor genes, amplification and/or mutational hyper‐activation of oncogenes.^30^ These genetic changes would greatly augment survival pathways and systematic defects in the apoptosis signalling machinery, resulting in resistance to anticancer therapies and contribute to relatively low effects to therapies. Recently, the increased incidences suggested that non‐apoptotic cell death modes such as induced autophagy, mitotic catastrophe, senescence and necrosis are becoming attractive targets for novel anticancer therapeutic drugs.^31^, ^32^ Indeed, autophagy can be a highly efficient mode of cell death induction by excessive self‐digestion in cancer cells or apoptosis‐deficient cells upon various chemotherapeutic drugs or radiotherapy.^33^ Notably, many chemotherapeutic drugs, including arsenic trioxide, pimozide, loperamide, amentoflavone, Cannabidiol and Celastrol, can trigger autophagy‐associated cell death in glioma cells.^34^, ^35^ Furthermore, targeting autophagy by CQ, dasatinib, PI103 greatly sensitives glioma to temozolomide (TMZ) treatment or radiation plus TMZ treatment and improve the therapeutic effects.^36^, ^37^ In the study, we present evidences to demonstrate that hirudin induces an autophagic cell death in GBM cells, but not apoptosis. Our results strongly suggested that hirudin could be utilized to improve glioma therapy by targeting the autophagic pathway.

Hirudin is the strongest natural specific inhibitor of thrombin found so far. Beside the potent anti‐thrombotic activity, additionally various functions of hirudin have been addressed as well, including anti‐tumour, anti‐fibrosis, anti‐hyperuricemia, wound repair, benefitting diabetic complications or cerebral hemorrhage^4^. These demonstrations address the fundamental importance of thrombin and hirudin in biology and medicine, even though the related mechanisms through which they regulate the physiological or pathological progression remain largely elusive. Recent data suggest elevated thrombin production not only increases blood coagulation, but also promotes tumour growth and metastasis.^38^, ^39^, ^40^ Tissue factor (TF)‐initiated thrombin generation is crucial for metastasis through metalloproteinase (MMP) 2 and 9 activation, fibrin and platelet deposition.^41^, ^42^ Thrombin‐dependent protease activated receptor (PAR) signalling is activated to upregulate the expression/secretion of integrin alphavbeta3, VEGF vascular endothelial growth factor (VEGF) receptors (KDR and Flt1), which are the markers of the angiogenic phenotype and cell proliferation.^43^, ^44^ These findings could explain the angiogenic and tumour‐promoting effect of thrombin and provide the basis for hirudin for anti‐cancer or other therapeutic application. In the study, we presented evidences to demonstrate that hirudin treatment causes an obvious decrease of mTOR signalling activity, which further results in a canonical ULK1‐LC3‐II‐dependent autophagy and growth arrest of GBM cells. Considering the existence of the signal transduction between PAR‐1/VEGF/PI3K/AKT pathway and mTOR signalling pathway,^45^ it could be inferred that hirudin‐induced LC3‐II expression and autophagy could be through the inhibition of thrombin and consequent suppression of the activity of PAR‐1/VEGF/PI3K/AKT/mTOR axis, in spite of the detail evidences need to be obtained further.

In conclusion, hirudin can induce autophagy‐dependent cell growth arrest through mTOR inactivation in glioma cells, but not apoptosis, which provides new insights into the molecular antitumor mechanism of hirudin and provides a promising strategy for the treatment of glioma patients.

## AUTHOR CONTRIBUTIONS

**Ying Ma:** Conceptualization (equal); data curation (lead); funding acquisition (equal); methodology (lead); software (lead); validation (lead); writing – original draft (lead). **Senbin Wu:** Methodology (equal); software (equal); validation (equal). **Fanyi Zhao:** Data curation (supporting). **Huifeng Li:** Data curation (supporting). **Qiaohong Li:** Data curation (supporting). **Jingzhi Zhang:** Supervision (supporting). **Hua Li:** Methodology (supporting). **Zhongmin Yuan:** Conceptualization (lead); funding acquisition (equal); supervision (lead); writing – review and editing (lead).

## CONFLICT OF INTEREST STATEMENT

The authors declare that the research was conducted in the absence of any commercial or financial relationships that could be construed as a potential conflict of interest.

## ETHICS STATEMENT

The animal study protocol was approved by the Animal Care and Use Committee of Guangzhou Medical University (A2021‐009, A2022‐053).

## Supporting information


**Supplementary Figure 1** The chemical structure of natural hirudin ^4^.

**Supplementary Figure 2** Anatomical images of CDX animal models treated with Hirudin. (A) Thoracic and abdominal images of CDX nude mice treated with 2 U/mL and 4 U/mL hirudin. (B) Cerebral images of CDX nude mice treated with 2 U/mL and 4 U/mL hirudin.

## Data Availability

The data that support the findings of this study are available from the corresponding author upon reasonable request.
