# Supplementary figures and images for: Hirudin inhibits glioma growth through mTOR‐regulated autophagy
Source: J Cell Mol Med. 2023 Aug 4;27(18):2701–13. doi: 10.1111/jcmm.17851 (PMC10494300; doi:10.1111/jcmm.17851)

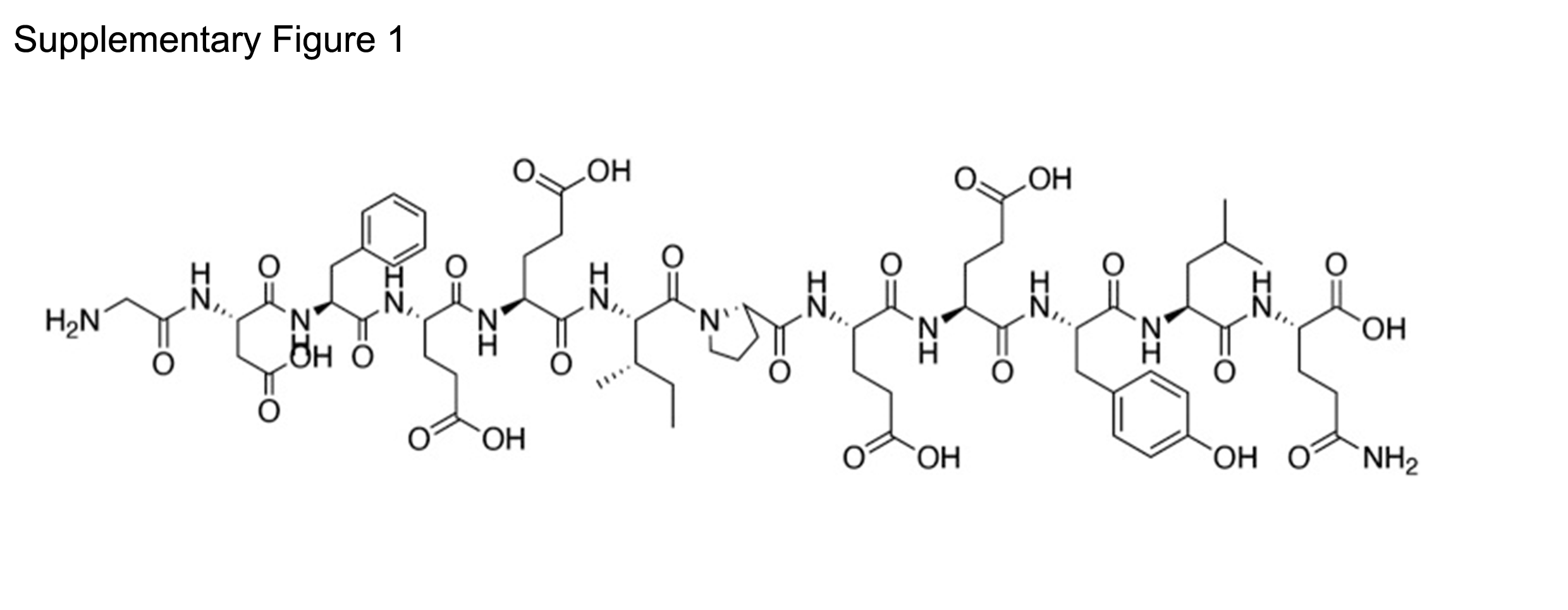

Supplement: Supplementary file 1 — Supplementary Figure 1 The chemical structure of natural hirudin 4. [file JCMM-27-2701-s001.tif]

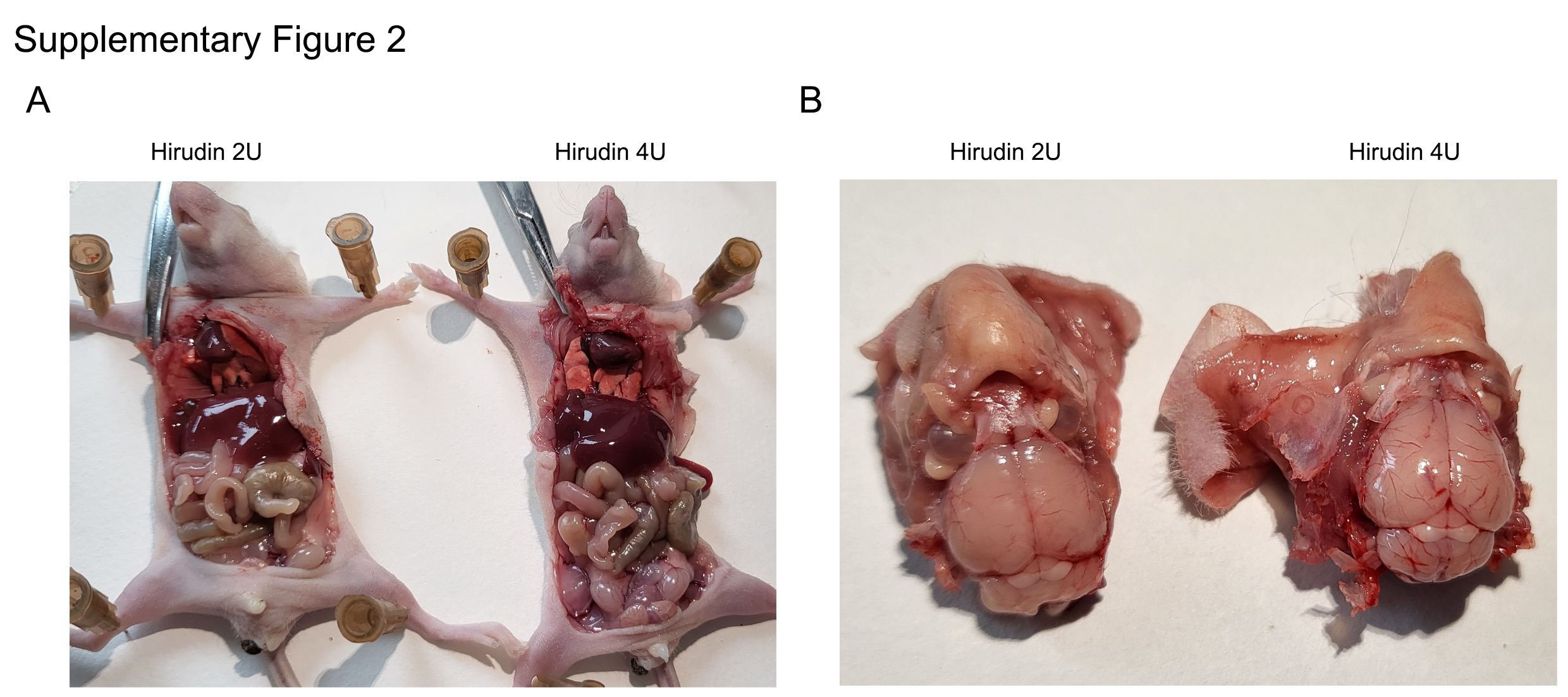

Supplement: Supplementary file 2 — Supplementary Figure 2 Anatomical images of CDX animal models treated with Hirudin. (A) Thoracic and abdominal images of CDX nude mice treated with 2 U/mL and 4 U/mL hirudin. (B) Cerebral images of CDX nude mice treated with 2 U/mL and 4 U/mL hirudin. [file JCMM-27-2701-s002.tif]
